# Operando Monitoring of Local pH Value Changes at the Carbon Electrode Surface in Neutral Sulfate-Based Aqueous Electrochemical Capacitors

**DOI:** 10.1021/acsami.2c09920

**Published:** 2022-08-10

**Authors:** Adam Slesinski, Sylwia Sroka, Krzysztof Fic, Elzbieta Frackowiak, Jakub Menzel

**Affiliations:** Institute of Chemistry and Technical Electrochemistry, Poznan University of Technology, Berdychowo 4, Poznan 60-965, Poland

**Keywords:** electrochemical capacitor, operando pH measurement, energy storage device, carbon electrode, neutral aqueous electrolyte, operando GC-MS measurement

## Abstract

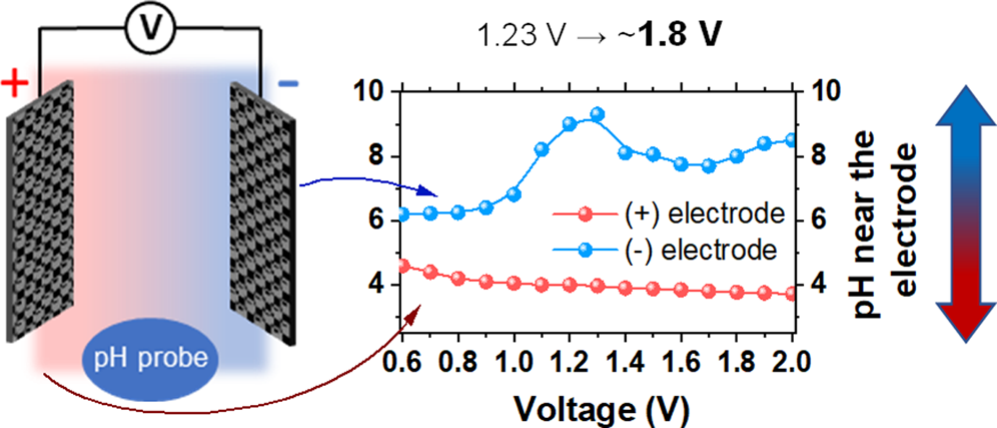

The operando monitoring of pH during the charging and discharging of an electrochemical capacitor in an aqueous neutral salt solution is presented. Proper knowledge of transient and limiting pH values allows for a better understanding of the phenomena that take place during capacitor operation. It also enables the proper assignment of the reaction potentials responsible for water decomposition. It is shown that the pH inside the capacitor is strongly potential-dependent and different for individual electrodes; therefore, the values of the evolution potentials of hydrogen and oxygen cannot be precisely calculated based only on the initial pH of the electrolyte. The operando measurements indicate that the pH at the positive electrode reaches 4, while at the negative electrode, it is 8.5, which in theory could shift the theoretical operating voltage well beyond 1.23 V. On the other hand, high voltage cannot be easily maintained since the electrolyte of both electrode vicinities is subjected to mixing. Operando gas monitoring measurements show that the evolution of electrolysis byproducts occurs even below the theoretical decomposition voltage. These reactions are important in maintaining a voltage-advantaged pH difference within the cell. At the same time, the electrochemical quartz crystal microbalance (EQCM) measurements indicated that the ions governing the pH (OH^–^) that initially accumulated in the vicinity of the positive electrode enter the carbon porosity, losing their pH-governing abilities. pH fluctuations in the cell are important and play a vital role in the description of its performance during the cyclability at a given voltage. This is especially noticeable in cell floating at 1.3 V, where the pH difference between electrodes is the highest (6 units). The increase of the electrode separation distance acts similarly to the introduction of a semipermeable membrane toward the increase of the capacitor cycle life. During floating at 1.6 V, where the pH difference is not as high anymore (4 units), the influence of separation in terms of electrode stability, although present, is less notable.

## 1 Introduction

**Figure 1:**
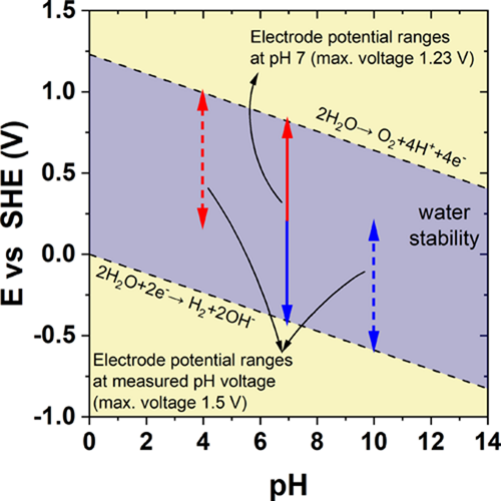
Pourbaix diagram for water.

The technological advancement and extensive production of electrically powered devices have led to the need to search for energy storage units produced on a large scale. Several widely used devices are powered by rechargeable units. Nowadays, electric vehicles (EVs), which are powered by lithium-ion batteries (LIBs), and related technologies in this field, have sparked a booming interest.^1−3^ This kind of battery can thus be successfully applied, owing to its high energy density, ensuring efficient driving range per charge, as well as other parameters such as weight ratio of the battery system to the whole car, etc..^4−6^ However, LIBs suffer from slow electrochemistry with strongly limited cyclability, which tremendously impacts their use in high-power applications, where fast acceleration or regenerative braking is of key importance.^7,8^ Additionally, they require long charging times and have limited cycle life due to volume changes in the material upon cycling operation, adding yet more drawbacks.^9−11^ The already well-known energy storage system that overcomes those issues is built with the use of electrochemical capacitors (ECs). The principle of operation of the EC differs strongly from that of an electrochemical cell, as it relies primarily on physical phenomena rather than chemical. In this way, the processes that occur are faster and much more reversible, allowing these devices to reach higher power density and superior cyclability (>10^6^ charge–discharge cycles).^12−14^ Owing to the charge storage mechanism, the voltage during capacitor charging and discharging ramps linearly with the state of charge, while for batteries it is almost constant, and its value depends on redox reactions. The major drawback of ECs is, without a doubt, their low specific energy storage capability. This problem can be tackled in two ways: by either increasing the operating cell voltage (*U*) and/or the specific capacitance (*C*), as these are the parameters that govern the energy stored in the device, as given by
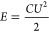
1In electrochemical capacitors, the choice of a solvent is of utmost importance, as it governs the maximum voltage of the system. Currently, capacitors that operate in an organic medium as an electrolyte solution, mainly based on acetonitrile or propylene carbonate as solvents,^15,16^ are the most widely used and commercialized, as the use of these media allows for a high, undisrupted voltage operation of up to 2.7 V.^17−20^ However, these solvents pose several important drawbacks such as high toxicity, flammability, and price, which can greatly hinder further implementations in both industry and research settings.^21−24^ The use of an aqueous medium proves to be a much better and greener alternative; however, it is characterized by a narrow voltage window of ∼1.23 V^25,26^ due to the decomposition of water, which, in turn, limits the energy stored by the device.^27−30^ Although numerous studies have been done on the topic, progress related to research based on the enhancement of the operating voltage of aqueous electrolytes is undoubtedly lacking, and much of the focus is directed toward the development of high-capacitance electrode materials.^31^ To increase operating voltage, potential ranges of both negative and positive electrodes must be considered, such as
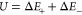
2(where *U* is the operating voltage, Δ*E*_+_ and Δ*E*_–_ being the potential ranges of positive and negative electrodes, respectively). However, these are strongly influenced by the oxygen evolution reaction (OER) and hydrogen evolution reaction (HER), respectively, which typically lead to a narrow stable thermodynamic potential window of aqueous electrolytes.^31^ Thus, considering this, if the cutoff value of Δ*E*_+_ is more positive and this of Δ*E*_–_ is more negative, a higher operating voltage could be achieved. From the practical point of view, to achieve high-voltage electrolyte, its pH must be considered, as shown in the Nernst equation
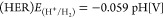
3
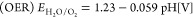
4where the theoretical electrode potentials of HER and OER can be calculated. Various approaches have been proposed to enhance this thermodynamically imposed limit. The most common is based on the differentiation of the pH of the solution in the vicinity of a single electrode. This is a vital concept, as the pH of the bulk solution can differ greatly from the pH at the surface of the electrode (as much as 4 pH units^32^) due to the consumption or production of H^+^/OH^–^ during electrochemical operation.^33^ Additionally, the differentiation of pH values at individual electrodes can be used to establish the maximum safe voltage of the cell. It can be visualized on the Pourbaix diagram as in Figure 1. In a fixed pH electrolyte, the electrodes can only operate safely within a limit of 1.23 V, while when the pH is differentiated, this voltage can be successfully enlarged.^34^ The effect of buffer agents on hydrogen adsorption and increase of pH at the interface were studied elsewhere; however, the pH was not measured in operando mode.^35^

So far, there have been numerous attempts to directly establish pH at the electrode surface from computational modeling and nonelectrochemical and electrochemical methods.^33,36−42^ Some of these methods have proven to be more successful than others. For instance, Fuladpanjeh-Hojaghan et al. have used a pH mapping technique in which the pH distribution in electrochemical processes was successfully measured at each electrode using laser scanning confocal microscopy and various pH-sensitive fluorescent dyes.^33^ It was shown that the pH at the positive electrode is lower (acidic) due to the presence of H^+^ and higher (alkaline) at the negative electrode due to OH^–^ formation. Other techniques employed, such as optical or some electrochemical methods, can prove inaccurate and prone to a vast number of errors ranging from factors such as experimental difficulties, costly and complex equipment employed, to the problematic nature of transient pH values during data collection.^32^

The results of the works presented above were promising; however, no solid evidence was given with a thorough study on the pH difference during electrochemical operation in a capacitor cell. Here, for the first time, we propose a simple, cost-effective, and accurate (within the margin of error of approximately ±0.1 pH value) operando pH monitoring at the vicinities of the electrodes in a capacitor setting, where pH value changes can then be quantified as a function of the applied voltage. This approach not only allows for an accurate determination of transient pH value changes upon polarization at a given electrode surface but also offers a fast and direct response with an exact pH value reading. This information can help to further expand the current knowledge and elucidate the role of pH changes, so that optimization and improvement measures on EC’s operation in aqueous media can be fulfilled.

## 2 Experimental Section

**Figure 2:**
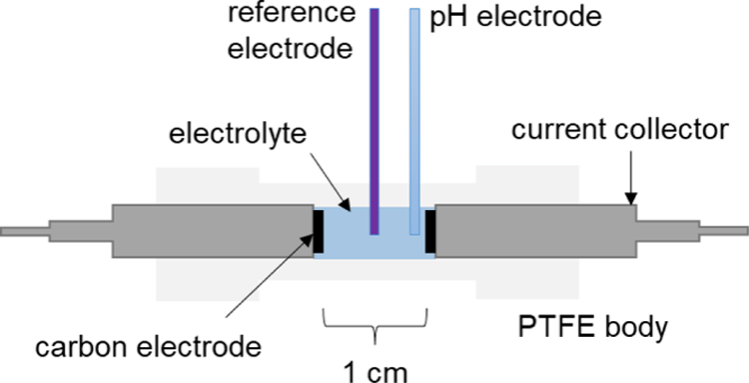
Scheme of the electrochemical setup and electrode positioning.

The electrochemical system consisted of binder-free carbon electrodes (Kynol ACC 507-20) of 10 mm diameter, anchored to the current collector using graphite conductive glue. The carbon electrode was of high purity, and additionally, temperature was treated at 120 °C for 2 h in a vacuum dryer to remove any physisorbed oxygen and water. Its final oxygen content was determined by direct elemental analysis to be 1.5%. They were soaked in a 1 mol L^–1^ electrolyte (Li_2_SO_4_, Sigma-Aldrich, >98%) solution. The simultaneous electrochemical and pH measurements were carried out in a PTFE-body (1/2 inch straight tube fitting union) two-electrode cell with stainless steel 316 L current collectors (Figure 2). The upper wall of the fitting was adapted with a longitudinal hole, where a pH sensor microelectrode and a reference electrode were introduced. The exact positioning of the pH sensing electrode depended on the specific measurement target and is precisely stated in the consecutive sections of the article. However, to fit two sensing tips, carbon electrodes were initially fixed at a 1 cm distance from each other. Certainly, it introduced additional ohmic resistance to the capacitor cell; however, testing the capacitor at a slow 5 mV s^–1^ scan rate or keeping it at constant polarization (floating) at 1.6 V allowed undisturbed results to be obtained. The reference electrode (Hg/Hg_2_SO_4_) was added to monitor the responses of individual electrodes for direct comparison with the pH readings. The experiments were carried out using a BioLogic VMP3 potentiostat/galvanostat equipped with an analogue operating amplifier for the acquisition of parallel pH values.

The evolution of gases during capacitor operation was monitored in PAT-Cell-Gas (El-Cell) in an online mode using gas chromatography-coupled mass spectrometry (GC-MS, Bruker). The monitored masses were *m*/*z* = 44 and 34, assigned to carbon dioxide and hydrogen peroxide, respectively.

The electrochemical quartz crystal microbalance (EQCM) investigation was conducted using 1 mol L^–1^ Li_2_SO_4_ solution (pH = 7.9) as an electrolyte. The thickness of Kynol makes EQCM studies not feasible; therefore, activated carbon (AC) YP-50F (Kuraray, Japan) was selected, having textural properties (micro:mesoporosity ratio) similar to Kynol ACC 507-20. As the main goal of EQCM in this article was to deal solely with ion adsorption and not the chemical reactions, its surface chemistry was not considered in this experiment. The electrodes, which were coated on the quartz crystal resonator, were prepared in 80:20 (w/w) for an AC:binder mass ratio. The slurry consisted of AC powder mixed with a 5 wt % solution of poly(vinylidenedifluoride) (PVDF) binder (Sigma-Aldrich) in *N*-methyl-2-pyrrolidone (NMP) solvent (Sigma-Aldrich). The suspension was evenly drop-casted on a quartz crystal resonator (standard frequency 9 MHz, SEIKO EG&G, Japan) with a stainless steel current collector (SUS304) and dried at 60 °C for 12 h. The mass loading of the carbon coating was kept within the range of 30–60 μg ensuring a thin and even layer. Electrochemical measurements were performed in a poly(ether ether ketone) (PEEK) cell designed for EQCM measurements. The working electrode was the AC-coated resonator, placed at the bottom of the cell. Stainless steel foil was used as a counter electrode, and a saturated calomel electrode (SCE, +0.241 V vs standard hydrogen electrode (SHE)) was used as the reference electrode. An excess of the electrolyte (400 μL) was added, avoiding the presence of bubbles.

Point of zero charge (pzc) was determined in the EQCM cell via staircase potential electrochemical impedance spectroscopy (SPEIS) (10 kHz to 1 mHz), for which the specific capacitance values were calculated at 1 mHz and then plotted against the varied applied potentials. This allowed the minimal value to be determined, representing pzc, that is, +0.2 V vs SHE.

Other electrochemical techniques included cyclic voltammetry (CV) at 50 mV s^–1^, to kick-start the electrochemical response, followed by 5 mV s^–1^, where the change in resonator frequency was simultaneously recorded by the EQCM and then recalculated into the change in mass with respect to the Sauerbrey equation
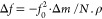
5where Δ*f* is the change in frequency, *f*_0_ is the fundamental resonance frequency of the crystal (Hz), Δ*m* is the change in mass (g), *N* is the frequency constant for a quartz crystal resonator (Hz Å), and ρ is the quartz density (2.648 g cm^–3^). Faraday’s law (eq 6) was used to recalculate data from CV, allowing one to compare the experimental and theoretical mass changes in the mass vs charge ratio plot
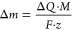
6where Δ*m* is the change in mass, Δ*Q* is the charge exchanged (C), *F* is Faraday’s constant (96 485 C mol^–1^), *M* is the molar mass of ion adsorbed/desorbed (g mol^–1^), and *z* is the number of exchanged electrons (i.e., the valence number of adsorbed/desorbed ions).

## 3 Results and Discussion

**Figure 3:**
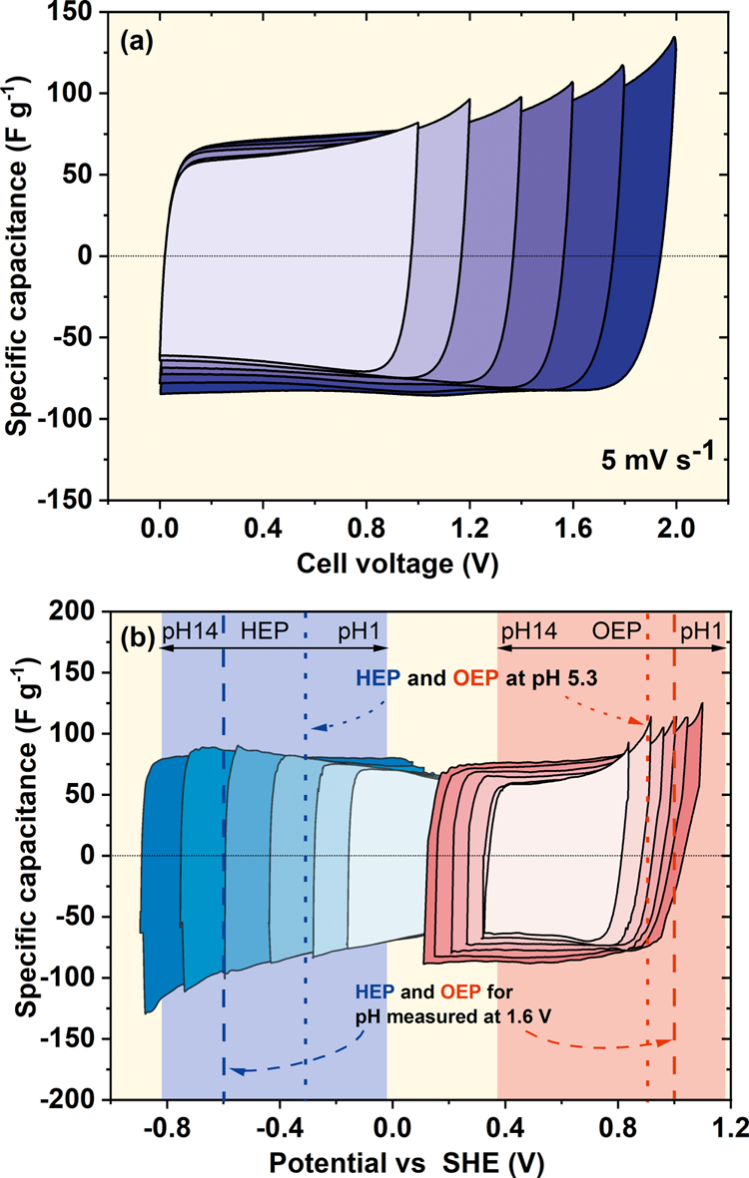
CV characteristics of ACC electrodes in the electrochemical cell (1 mol L^–1^ Li_2_SO_4_) during voltage extension from 1.0 to 2.0 V: (a) full cell and (b) individual electrodes. The shaded regions indicate the possible HER and OER regions in the full pH range; dotted lines indicate the theoretical solvent decomposition potential; dashed lines indicate the graph read potentials.

Positioning the electrodes 1 cm apart was done not only to accommodate the sensing electrode tips but also to ensure that the electrolyte at the electrode vicinities was not under the mixing conditions, so that the experiment could be carried out in a controlled manner. Indeed, it might seem that such a configuration differs from the real capacitor cell, where the electrodes are tightly squeezed with the separator in between; however, for the demonstration, proof-of-concept, and further optimization purposes, this configuration was selected. Figure 3a shows cyclic voltammograms taken at 5 mV s^–1^ during the voltage window extension from 1.0 to 2.0 V, with a 0.2 V step. Even though a voltage of 2.0 is considerably high for the nonmodified system in the aqueous-based electrolyte, such a limit was selected to observe the effects within an extended range. Figure 3b contains the electrochemical response of the electrodes, which was recorded in the presence of a reference electrode. It is clear that after exceeding the thermodynamic stability of the solvent (water), the current leaps, related to its decomposition, appear. As the water ions take part in the reactions, the potentials at which the reactions take place are pH-dependent. The shaded regions indicate the limiting area where the H_2_O decomposition starts within the entire pH range of 0–14.

It is possible to calculate the theoretical equilibrium potentials of the reactions of water reduction and oxidation at a given pH based on the formulae in eqs 3 and 4. The initial pH of the electrolyte is 7; however, after contact with carbon electrodes, it changes to 5.3. This immediate change occurs due to the presence of surface functional groups. Assuming that it remains unchanged during operation, the above potentials should be equal to *E*_red_ = −0.31 V and *E*_ox_ = 0.92 V, respectively (dotted lines). In contrast to the just-mentioned theoretical values, it is possible to read out the potential of the reactions of reduction and oxidation directly from the graph if it is assumed that the onsets of equilibrium reactions are exactly at the potentials where the faradic current starts to increase exponentially. Here, they are estimated to be −0.6 and 1.0 V, respectively (dashed lines). The discrepancy in the potential values emerges solely from the shift of equilibrium potentials, being dependent on the proton concentration polarization. If these potentials are recalculated into pH values using the rearranged version of eqs 3 and 4, they will correspond to pH values of 10 and 4.6, respectively. In the case of the negative electrode, the pH is higher by almost 5 units, while in the case of the positive electrode, the difference is not that significant. Furthermore, as might be observed in Figure 3a,b, the sharp increase in the current values recorded at the end of the charging sweeps becomes progressively postponed along with the extension of the voltage, suggesting that the pH values are constantly modified, thus dependent on voltage. Therefore, marking the vertical lines that indicate the onset of decomposition reactions as in Figure 3b (dotted, theoretical) makes sense only when the pH is known at a given point of potential. It is not enough to stipulate them only prior to the cell operation. Proper determination of the theoretical solvent decomposition line locations can be done only if the pH is known, that is, the solution is fully buffered or the pH is measured in parallel.

**Figure 4:**
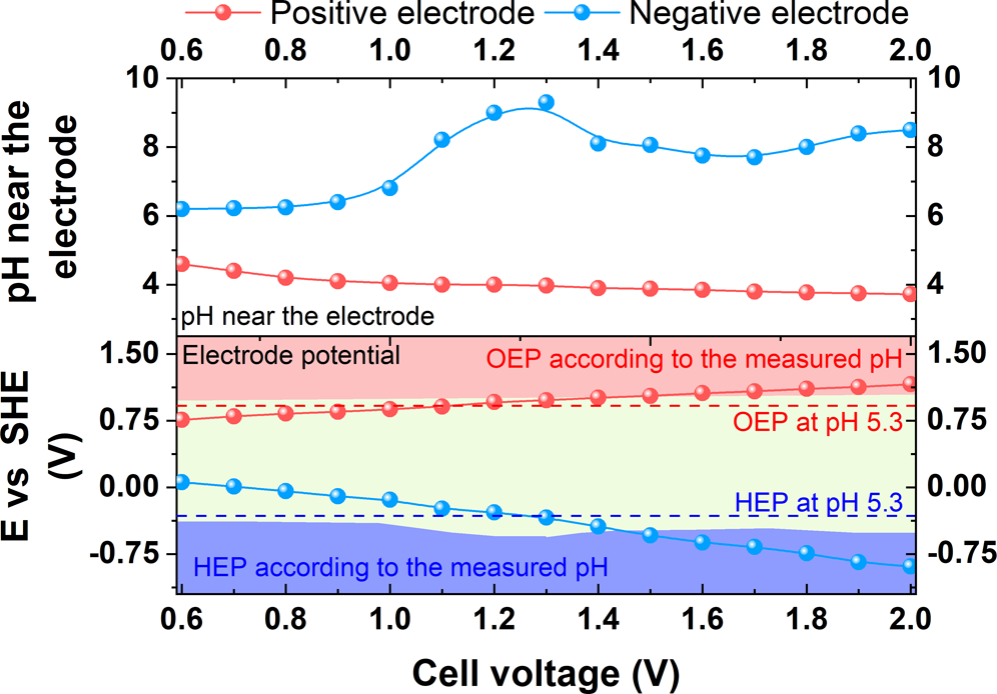
Upper graph: electrolyte pH at the electrode surfaces of the capacitor charged to different voltages in 1 mol L^–1^ Li_2_SO_4_. Lower graph: measured electrode potential values and corresponding HEP and OEP at these pH values. The dashed lines indicate the theoretical OEP and HEP at a constant pH.

As the pH of the neutral salt solution is susceptible to changes, the introduction of a pH sensing probe into the capacitor cell was necessary to indicate the pH at a given time (potential). The measurement of pH is, of course, the same as the measurement of the concentration of H^+^ ions; therefore, to ensure correct indications, the capacitor charging/discharging rates had to be slow enough to account for ion diffusion within the bulk of the electrolyte. This is because the ion adsorption/desorption phenomenon at the electrode/electrolyte interface is faster than the ion diffusion within the capacitor cell volume. Figure 4 shows the electrode potential extrema along with pH readings during the potentiodynamic voltage extension of the capacitor from 0.6 to 2.0 V with a 0.1 V step.

**Figure 5:**
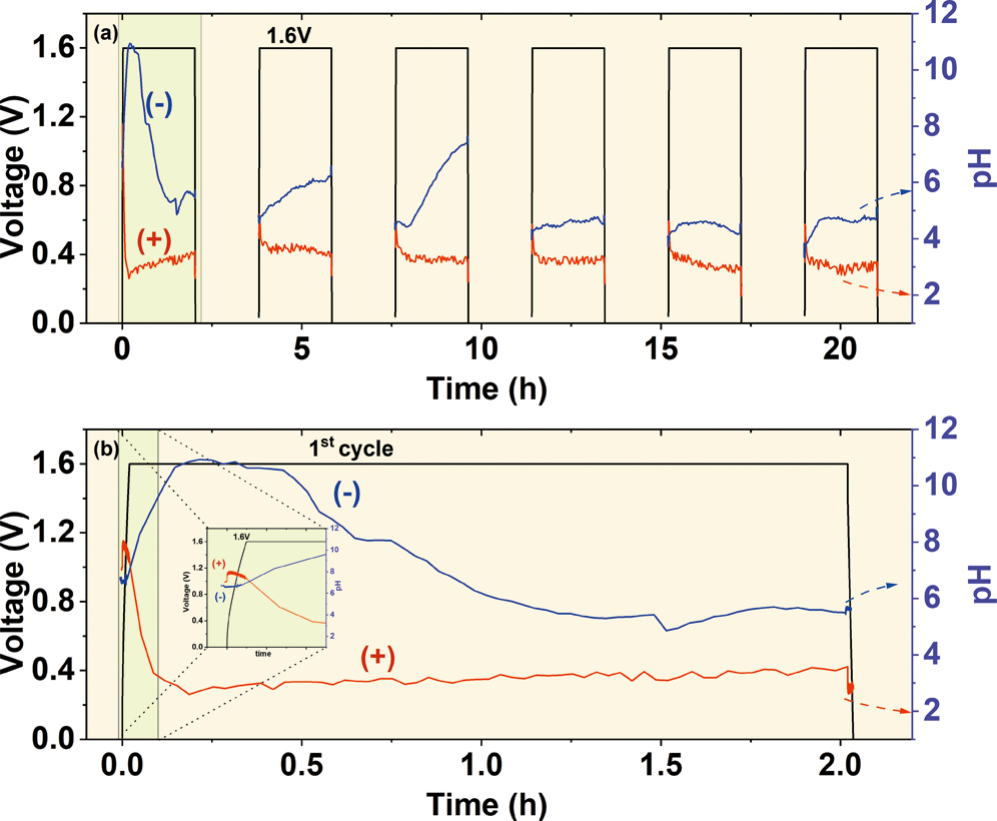
pH values monitored during potentiostatic floating at 1.6 V during (a) six potentiostatic cycles and (b) first cycle with the inset into the charging period. The pH values of both electrodes are represented on the secondary *y*-axis.

It was noted that the pH at the positive electrode experienced progressive acidification. This effect was more evident at lower voltages, and then it decelerated when the voltages were above 1.0 V. It was probably balanced by the visible pH hump at the negative electrode, which started at that point of voltage, which is discussed below. The deceleration is therefore related to difficulties in maintaining the concentration polarization, as the pH was further from its initial value. Namely, once the cell voltage reached 1.0 V, the pH at the positive electrode dropped from the initial value of 5.3 down to 4, but further (from 1.0 to 2.0 V) only slightly to around 3.6. However, one should remember that the pH is a logarithmic unit and the real concentration changed significantly. The overall behavior of the pH at the negative electrode is more complex. First, the pH at 1.0 V was almost the same as the initial pH of the electrolyte. The total change did not follow a one-direction upward trend. Instead, the visible and significant change started at 1.0 V of cell voltage. It reached maximum alkalinity of 9.5 at around 1.3 V and then slightly declined to 8 at 1.7 V. Above 1.7 V, it increased back again to reach 8.5. Considering the pH balance within the system, the irrational change at the negative electrode can have its explanation in the analysis of accompanying electrode reactions. Second, it may be deduced that the pH at the negative electrode is strongly influenced by the concentration impact of the positive electrode or, in other words, the positive electrode domination. After a comparison of pH behavior at both electrodes, it can be seen that the maximum pH difference was 5 at a voltage of 1.3 V. Whenever there is a change in pH, the OER and HER potentials change. The pH measurements allowed for the actual HEP and OEP calculations to be performed (eqs 3 and 4). The calculated HEP and OEP are therefore corrected by the factor of variable pH (the results are shown as shaded regions in Figure 4). When the theoretical potentials of HEP and OEP are compared with the assumption of fixed pH (dashed lines) with the potentials of these reactions at instantaneous pH measured at a given cell voltage, the increasing discrepancy is noticed. Taking into account the terminal electrode potentials and the electrode reaction potentials (HER and OER), the safe cell voltage limit appears to be 1.4 V. Although the probe tip was placed as close to the surface of the carbon electrode as possible, the distance was still higher than the diffusion layer thickness. Different scan rates of the experiment could result in different pH readings. For example, lower scan rates (which give more space for electrode kinetic-related effects to occur) would provide a situation that is closer to potentiostatic voltage hold conditions. Higher sweep rates would result in incorrect readings due to cell resistance overpotential. Therefore, to increase the data reliability, accounting for concentration polarization and slight variations of pH during alternate charging and discharging, it was decided to measure the pH under conditions resembling the stationary ones. Accordingly, it was done under potentiostatic conditions after the leakage current leveled out, therefore, when the concentration gradient reached equilibrium. Figure 5 shows pH readings during potentiostatic conditions at a cell voltage of 1.6 V. Again, looking at the overview (Figure 5a), the pH values are more stable at the positive electrode, exactly as it was observed during potentiodynamic experiments. Moreover, they are relatively stable within a single floating period of 2 h (excluding charging and discharging periods). On the contrary, the pH at the negative electrode exhibited more fluctuations with time and cycle number. Once the cell was charged to 1.6 V, the pH at the negative electrode immediately changed to the alkaline area (pH = 11), while that at the positive electrode was changed to the acidic one (pH = 3), as observed in Figure 5b. Such a pH difference was kept for a short time, and after less than half an hour, the pH difference diminished due to the loss of alkalinity at the negative electrode. At the same time, the pH of the positive electrode remained at the same value. This was yet another proof that the positive electrode imposed the negative one and finally governed the pH within the entire capacitor cell. It is easily seen in subsequent cycles that the pH of the negative electrode tends to increase; however, it is probably suppressed by the impact of the positive electrode. The decrease in pH difference could probably be responsible for the reduction in future cell lifetime. The interesting pH fluctuation is observed within the first seconds of cell first charge, where the pH at the positive electrode increases, while the pH at the negative electrode decreases (Figure 5b, inset).

**Figure 6:**
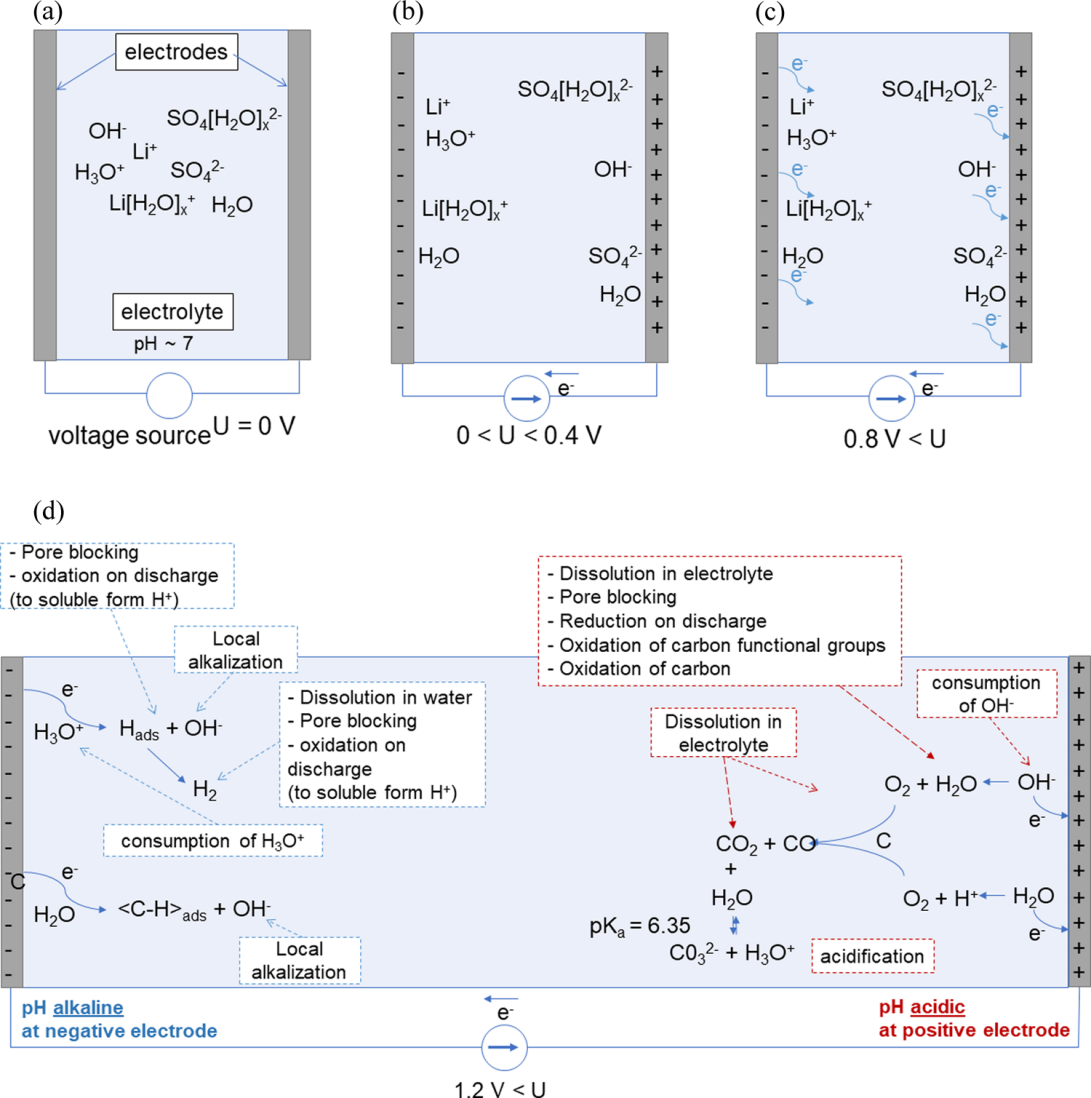
Schematic representations of electrode/electrolyte interfaces: (a) no polarization, (b) low voltage, (c) high voltage, and (d) selected chemical reactions taking place at high voltage.

The detailed explanation of pH variation at the electrodes can be understood when considering the movement of ions and reactions taking place at the electrode/electrolyte interfaces. To provide a clear explanation, three scenarios should be considered: in no cell polarization (*U* = 0 V), in low-voltage polarization (0 < *U* < 0.4 V), and in high-voltage polarization (*U* > 0.8 V). These are shown schematically in Figure 6. Of course, when no polarization is applied, the ions are randomly distributed within the electrolyte solution (Figure 6a). When the voltage is progressively increased, the ions tend to move according to the action of the electric field, i.e., the positively charged cations move to the negative electrode, while the negatively charged ions move toward the positive one. Accordingly, the positive electrode compartment should undergo alkalization, while the negative one undergoes acidification. This statement finds confirmation, as observed previously in Figure 5b (inset), where it is indeed true initially during charging at low polarizations. The situation changes when the potential builds up further and dielectric breakdown of the electrode/electrolyte interface begins to occur. Electrochemical reactions owing to the electron transfer impose the equilibria and, in turn, the pH at the electrodes. Again, it can be observed in Figure 5b (inset), where the direction of the pH changes immediately in the opposite direction.

The chemical reactions responsible for the government of pH are listed in Table 1. Contrary to planar and noble electrodes, the typical water decomposition electrode reactions are modified; here, porous carbon electrodes can be partly consumed. The general products are chemisorbed (<C–H>_ads_) or gaseous hydrogen (H_2_) at the negative electrode as well as CO_2_ or modified surface functionalities at the positive one. As demonstrated, not only can the neutral form of water (H_2_O) react as in reactions 9, 14, and 16 but also its charged and protonated form, H_3_O^+^ (reaction 10), and the deprotonated form, (OH^–^) (reaction 15). The possible evolution of gaseous hydrogen (H_2_) occurs at higher overvoltages at the negative electrode (reaction 11) or by the consumption of the oxygen groups from the carbon surface groups (reaction 12). The second case is confirmed by the reduced oxygen content in the negative electrode composition after the accelerated aging test at high voltage.^43,44^ H_2_O_2_, on the other hand, a common byproduct of water electrolysis, which takes place at the positive electrode (reaction 17), can be further decomposed into water and oxygen. Oxygen will immediately react with carbon through two processes: in the first, CO_2_ is produced (reaction 18); in the other, oxygen is chemisorbed. It has been suggested in the literature that H_2_O_2_ produced on the positive electrode might have a tendency to migrate toward the negative electrode through a separator, where it is consumed.^45^ This would explain the evolution of CO_2_ from reaction 13 at the negative electrode. Certainly, the same H_2_O_2_ decomposition reaction would take place as in reaction 18 at the positive electrode. Consumption of H_3_O^+^ and simultaneous production of OH^–^ intensify the alkalization rate at the negative electrode. This is why the hump at the negative electrode pH is observed in Figure 4. At sufficient overvoltage, reaction 12 takes place and further alkalization is suppressed. An important observation can be made by combining the substrates of reaction 15 with the products of reaction 16
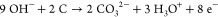
7This reaction in eq 7 can explain two phenomena: the disappearance of the alkaline character at the positive electrode when increasing the voltage above 0.4 V (consumption of OH^–^ as high as 9 moles, therefore its strong acidification) and production of the solid-state deposit of lithium sulfate according to the reaction in eq 8(43)
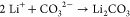
8All of the electron-transfer reactions mentioned in Table 1 are not visible as typical peaks on voltammogram (Figure 3), but rather as current leaps because the substrates for these reactions are different forms of a solvent, which are constantly available within the diffusion layer of the electrode/electrolyte interface.

**Table 1:** Electrode Reactions that Impose the pH at the Interfaces

negative electrode	positive electrode
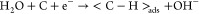 9	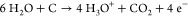 14
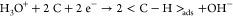 10	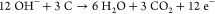 15
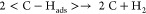 11	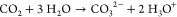 16
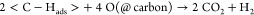 12	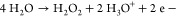 17
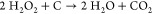 13	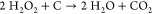 18

**Figure 7:**
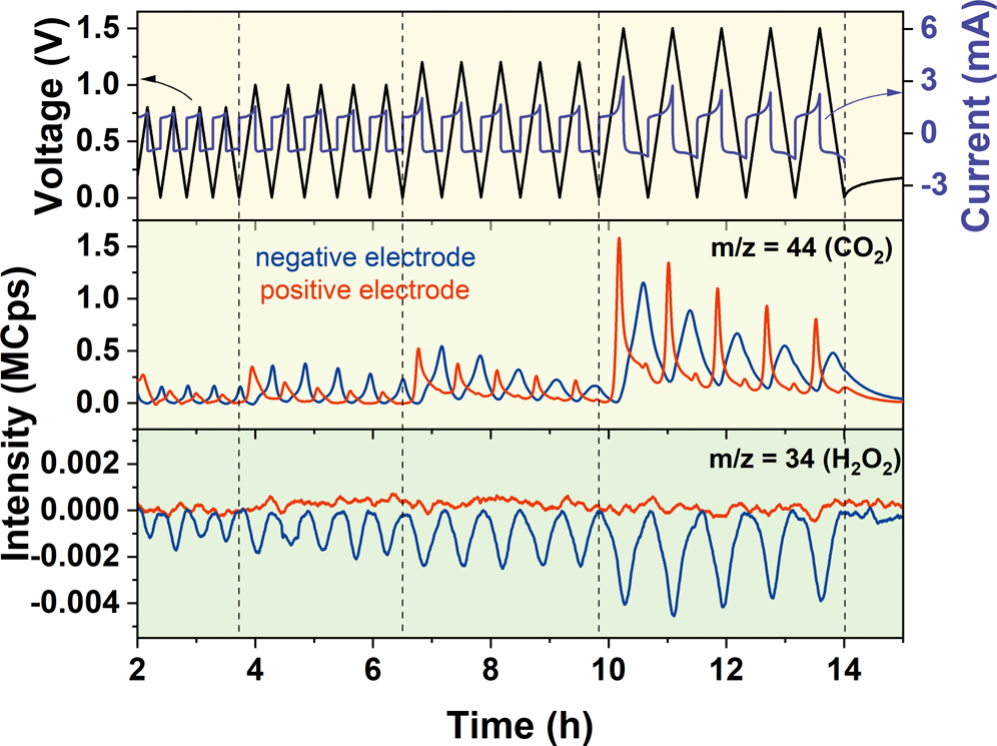
CO_2_ evolution pattern is a fingerprint of the presence of the water decomposition reaction. The first row shows the electrochemical data, while the other row shows the evolution of carbon dioxide and hydrogen peroxide.

It was experimentally proven using the GC-MS experiment that the chemical reactions listed in Table 1 take place under these conditions even below the theoretical limit of water decomposition (1.23 V). The evolution patterns of CO, CO_2_, and O_2_ gases were already presented elsewhere; however, they were not fully discussed.^46^ This seems possible in the case where the internal pH difference within a capacitor cell is against the voltage-advantage direction; the positive electrode operates in the alkaline region, and the negative electrode operates in the acidic region (Pourbaix diagram), which is directly correlated with the ion separation pattern shown in Figure 6b. This is the reason why the evolution of CO_2_ is observed even at a voltage as low as 0.8 V (Figure 7). The delay in gas release at the negative electrode is observed due to gas ejection during discharge at lower voltages. The evolution of CO_2_ at the negative electrode can be found owing to reactions 12 and 13. The presence of CO_2_ at the negative electrode may also be a consequence of gas diffusion in the capacitor cell during measurements. The signal of *m*/*z* = 34 (H_2_O_2_) was detected only at the negative electrode in the form of consumption peaks. This confirms that reaction 13 is valid, and, rather, all of the H_2_O_2_ produced on the positive electrode migrates from the positive electrode toward the negative one. The unfavorable conditions emerging only from the electrostatic attraction (low pH difference) are therefore counteracted by the chemical reactions of water decomposition. It means that the increase of capacitor voltage becomes a self-perpetuating phenomenon. This behavior suggests that the adverse pH difference formed beforehand at low voltages is responsible for the limitation of the stability window (<1.23 V), while it progressively broadens as the voltage increases.

**Figure 8:**
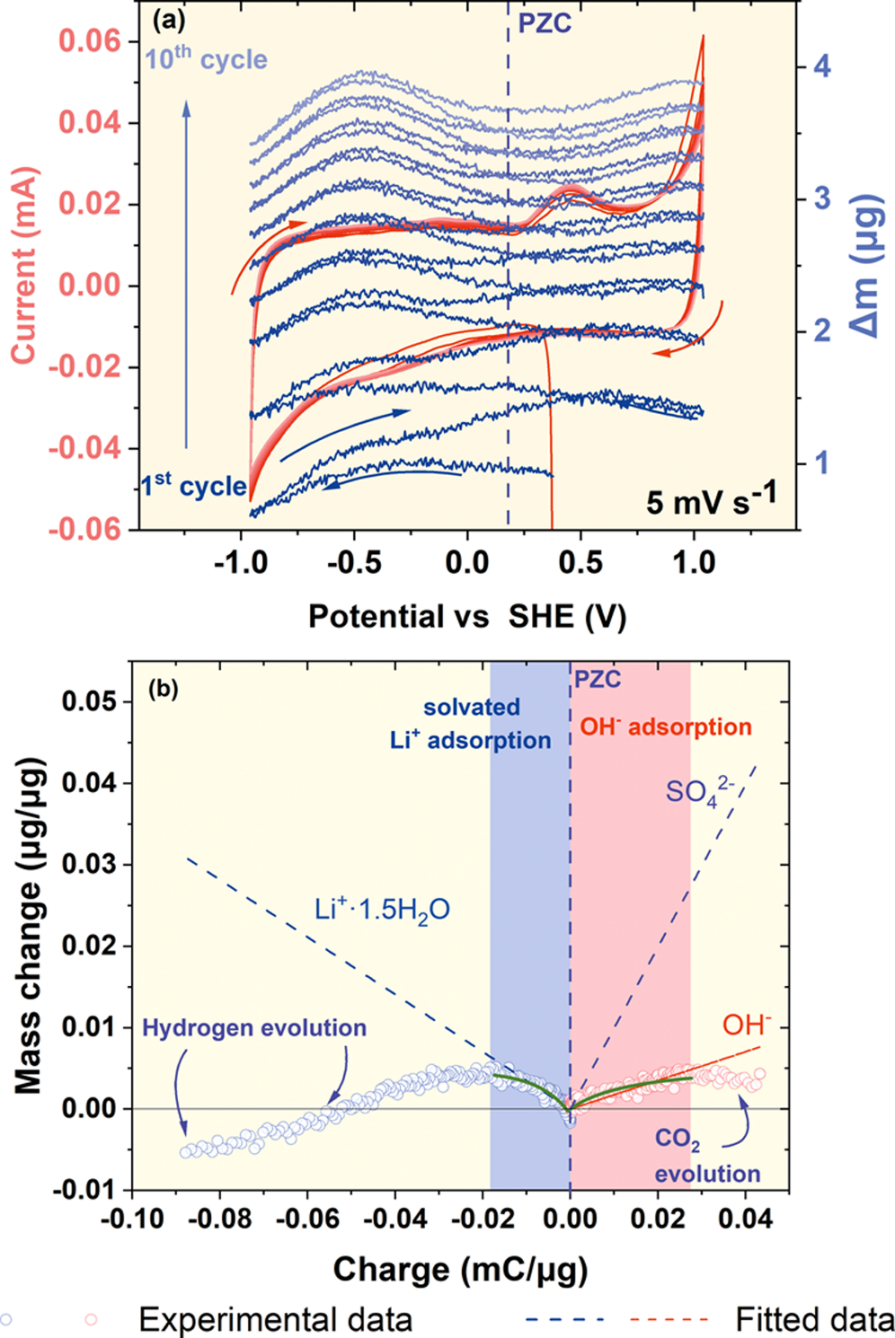
(a) CV and mass change response of YP-50F in a 1 mol L^–1^ Li_2_SO_4_ aqueous electrolyte at 5 mV s^–1^ and (b) electrode mass change vs charge during the polarization.

To confirm the presence of the mentioned ions, EQCM measurements were conducted. This technique provides information about the ion and solvent molecule fluxes during the charging process of the porous AC electrode. The mass change profile and current response recorded for YP-50F operating in 1 M Li_2_SO_4_ electrolyte at 5 mV s^–1^ are presented in Figure 8a, where a similar trend in mass change and electrochemical response is observed for each cycle, proving its repeatability. Because of the low carbon loading on the resonator, the system is more sensitive to redox reactions and electrolyte decomposition. The sharp change in the current response observed at the terminal potentials is linked to electrolyte decomposition reactions associated with hydrogen storage and oxygen (carbon dioxide) evolution. Figure 8b shows the charge-to-mass ratio plot, with theoretical and experimental curves calculated from the Faraday law. The slopes fitted to the experimental curves were found to correspond to the adsorption of Li^+^·1.5 H_2_O and OH^–^ for negative and positive polarizations, respectively. As it is not possible to adsorb 1.5 molecule of water, it is an average number of water molecules coadsorbed with the lithium ions. The exact regions for Li^+^ and OH^–^ adsorptions are denoted in the highlighted area on the graph. To eliminate the possibility of adsorption of other anions, a theoretical curve for bare [SO_4_]^2–^ was calculated, as seen in Figure 8b. The slope clearly does not correspond to the experimental one, debunking that possibility.

The EQCM results confirm the assumptions previously stated for the pH changes observed on the positive electrode in Figure 5. Initially, the pH value increases, as there are more OH^–^ species available near the electrode surface. With an increase in potential, the adsorption of OH^–^ takes place (some of which adsorb in the pores); thus, less OH^–^ is present in the vicinity of the electrode. Simultaneously, the adsorbed OH^–^ ions are consumed as in reaction 15 in Table 1. This brings about acidification, which lowers the pH at the positive electrode.

Upon negative polarization from pzc, an increase in the mass of the electrode can be observed, which denotes the adsorption of the cations, with insignificant change in the population of anions. A sharp decrease in mass can then be observed, which might be related to (1) the simultaneous adsorption of bare Li^+^ and repulsion of OH^–^ (*M* = 6.941 and *M* = 17.008 g mol^–1^ for Li^+^ and OH^–^, respectively), (2) repulsion of the water molecules from the bulk of pores and simultaneous adsorption of solvated ions, (3) desolvation of the Li^+^ ion, or (4) evolution of gas bubbles, which can negatively impact accuracy in the measured EQCM response.

For positive polarization, the electrode mass was found to increase slightly, which denotes the adsorption of bare OH^–^. Then, a decrease in mass is observed (as for negative polarization), indicating similar behavior, where most likely (1) a large amount of water molecules are expelled from the pores while OH^–^ is adsorbed, (2) OH^–^ is adsorbed and solvated Li^+^ gets expelled, or (3) the presence of carbon dioxide gas bubbles.

**Figure 9:**
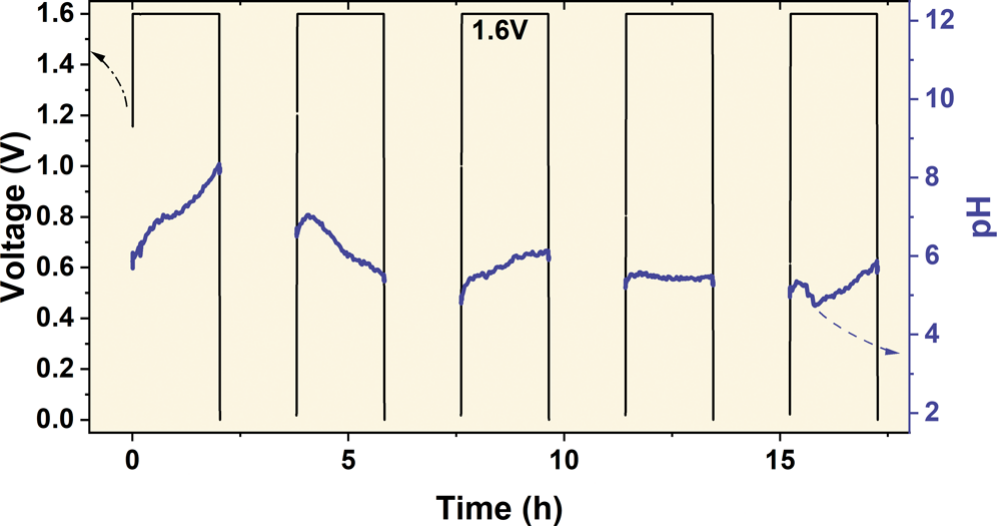
pH value monitored during potentiostatic floating at 1.6 V in 1 mol L^–1^ Li_2_SO_4_, with the electrode distance reduced to 4 mm in a pH cell.

The pH fluctuations were also monitored for the capacitor in which the carbon electrodes were positioned as close as possible (4 mm from each other) and the pH sensing probe was at the same distance to both electrodes, thus exactly in the middle. Figure 9 shows the results of the pH monitoring during potentiostatic floating at 1.6 V. In this case, the pH varies strongly between 4.8 and 8.1. Initially, the electrolyte becomes alkaline, yet in the second cycle, it becomes acidic. This behavior is probably found in real electrochemical capacitors, where electrolytes from both adjacent carbon electrodes mix easily, which in fact overlap and are subject to mixing. Here, another proof of positive electrode acidic environment domination is found.

The measurement of pH values at the electrodes might be helpful for the determination of the maximum safe voltage of the cell at which the electrode potentials remain below the solvent decomposition onset potentials at given pH values. As the pH difference does not increase proportionally with voltage (which can be deduced from Figure 4), there is an optimum voltage value, at which the pH difference is the highest. In the case of the investigated system, it is 1.3 V. This value is close to the theoretical value of water decomposition, with the additional contribution of hydrogen overpotential related to (owing to alkalization at the negative electrode) creating the optimum environment for the high cyclability of the cell. This may also contribute to the reduced gas production within the aqueous capacitor cell, which normally occurs at high voltages. The current methodology provides reliable verification of any pathway aimed at the introduction of the pH difference.

**Figure 10:**
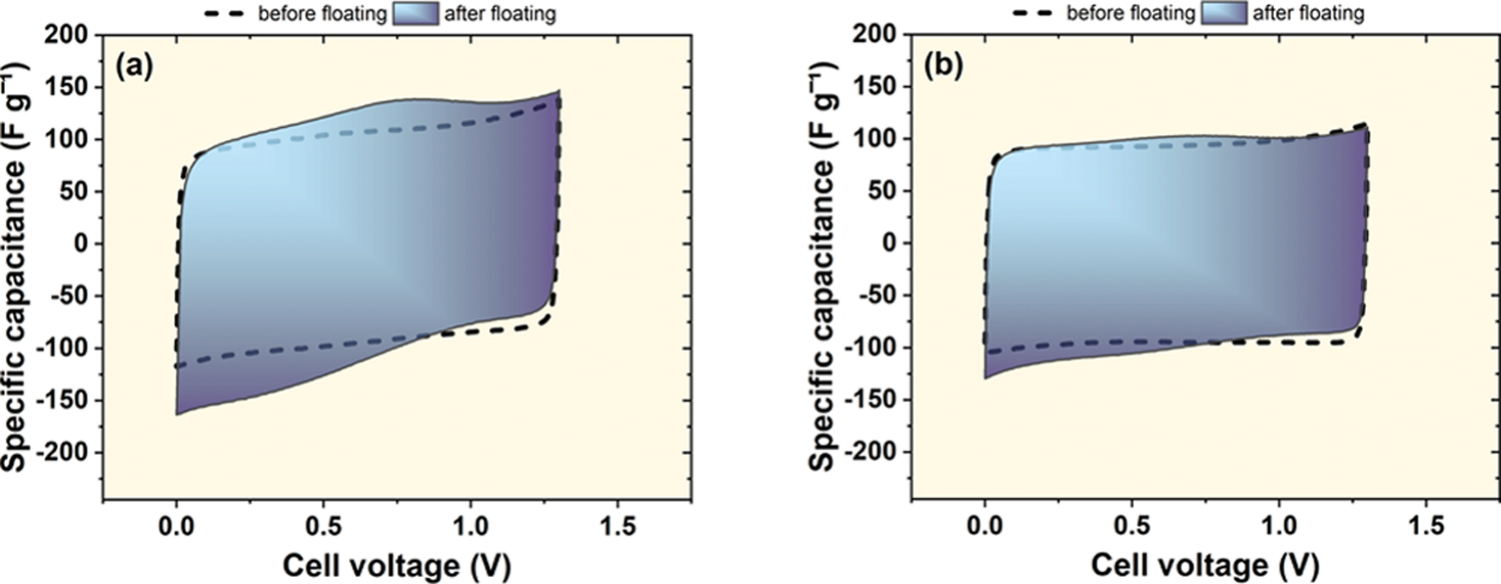
Cyclic voltammograms of the capacitors in 1 mol L^–1^ Li_2_SO_4_ recorded before and after the floating experiment at 1.3 V (5 mV s^–1^). Capacitor containing (a) one separator and (b) three separator layers.

As the results of the investigations in the test cell indicate that the distance between the electrodes influences the pH gradients, it has been decided to verify the concept in the real electrochemical capacitor. For that purpose, the Swagelok cell was used and modulated electrode distances were applied. To be precise, it was done by employing one or multiple (three) separator layers. As the pH gradient was strongly influenced by the maximum cell voltage, the behavior was investigated at two voltages, that is, at 1.3 V (where pH difference is as high as 6 units) and 1.6 V (where pH difference is 4 units). Figure 10 shows the comparison of the cyclic voltammograms recorded before and after floating for 60 h at a voltage of 1.3 V using different separation distances of the electrodes. Both systems experienced aging after this floating experiment—the current humps suggesting a reversible reaction took place and the current leaps at the end of discharge were observed. The first case is always observed during floating at an elevated voltage and is related to oxidation of the positive electrode.^43,47−49^ The latter case is related to the desorption of hydrogen at the negative electrode, which was produced during the high negative polarization of the electrode.^50,51^ The aging phenomena are more evident in the cell where there was only one separator, that is, where the electrodes were closer to each other. It means that the electrodes positioned this way were aggravated by the mutual influence.

Visibly, the energy efficiency before and after the test dropped much more in the case of one separator system. All of these additional faradic processes contribute to the pseudocapacitance in the system. It is not desirable as it is related to the aging of the system. The drop in discharge capacitance at high voltages is equal in both cells, probably related to a phenomenon not related to pH fluctuations in the cell but rather due to pore clogging.^43,44,47^

**Figure 11:**
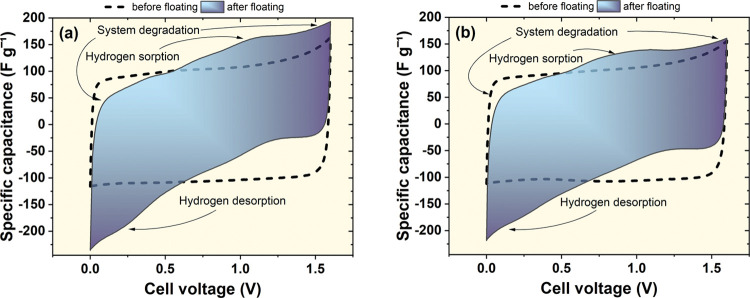
Cyclic voltammograms recorded for capacitors in 1 mol L^–1^ Li_2_SO_4_ recorded before and after the floating experiment at 1.6 V (5 mV s^–1^). Capacitor containing (a) one separator and (b) three separator layers.

A similar floating experiment was also conducted for a voltage of 1.6 V. In fact, the aging was more pronounced than in the case of 1.3 V. Depending on the separation, differences might be noticed (Figure 11). This time, they are slightly less visible than in the case of 1.3 V; however, the tendency remained similar. Again, three separator systems retained better performance. After floating at 1.6 V, extensive deterioration is observed during discharge. Capacitance at high voltages is diminished, while additional capacitance appears at low voltages. This is caused by the fact that each polarized electrode cannot further retain its beneficial pH conditions due to the internal mixing of the electrolyte. Again at 1.6 V, the energy efficiency before and after the test dropped much more in the case of one separator system.

The process of water decomposition appears to be self-limiting in nature (acidification at the positive electrode and alkalization at the negative one); however, if these two processes are not exactly balanced, one may dominate and affect the other one. It seems to be extremely difficult to maintain the balance; therefore, a straightforward voltage increase above 1.3 V in aqueous-based capacitors having nonmodified electrodes is difficult. The adequate modification of electrodes, electrolytes, separators, or a combination of all of those seems to be necessary.

An important aspect inherent to the regulation of separation distance is, of course, the resistance of the electrolyte. As the electrolyte resistance contributes the most to the total series resistance of the cell, it might be anticipated that the higher the distance (thicker separator), the higher the series resistance. High-frequency impedance measurements indicated that the series resistance (equivalent series resistance (ESR)) of cells containing one and three separators increased twice from 0.45 to 0.92 Ω. Certainly, together with distance, the electrolyte reservoir volume is different. However, the total electrolyte volume between the electrodes does not play a crucial role at 1.6 V polarization. First, the electrolyte is subjected to a concentration gradient during polarization of the cell, and second, the electrolyte solvent progressively decomposes when it experiences excessive voltage. The former seems not to be an issue since the concentration of the electrolyte is usually high (at least 1 mol L^–1^) and ion depletion would not occur. The latter can lead to electrolyte solvent depletion, an increase in the electrolyte salt concentration, and then its precipitation after the solubility limit is reached. The additional electrolyte solution reservoir in the carbon porosity might be an important property of the carbon, which can allow for a controlled environment in the system and thus becomes handy for the optimization of cell voltage abilities. The separation of electrodes (and their surrounding electrolyte) can also be done using a membrane. It was shown that it can successfully extend the cycle life. For comparison, the electrolyte content in organic-based capacitors found in commercial units is extremely low (the separator can be as thin as 30 μm). At the same time, they do not require this kind of consideration, as the organic electrolyte ensures a satisfactory voltage to be reached, without exhibiting electrolyte decomposition redox reactions. It puts more effort on aqueous-based capacitors to be designed; however, by considering all of the above-mentioned aspects, it can be equally efficient. Together with its environmental and economic impact, it is certainly a noteworthy challenge. For the justification of carbon electrode role in the behavior of pH changes, a measurement with bare stainless steel current collectors was done. As a result, the pH at both stainless steel electrodes increased. The increase of pH at the positive electrode was a result of OH^–^ ion attraction, while the alkalization at the negative electrode occurred due to discharging of the hydronium ions (H_3_O^+^) decreasing their concentration. As stainless steel is generally resistant to corrosion in these relatively short time measurements, its impact was not detected and studied. The long-term study of corrosion effects is interesting and important, which opens a new space for experiments.

## 4 Conclusions

The operando monitoring of local pH value changes at carbon electrode surfaces in aqueous electrochemical capacitors has been presented. The use of joint research techniques (pH operando monitoring, GC-MS, and EQCM measurements) allowed for a full description of the ion fluxes and electrolyte pH changes in an aqueous electrochemical capacitor.

In general, electrochemical capacitors with Li_2_SO_4_ solution used as an electrolyte are recognized as “neutral aqueous capacitors”. Our research shows that the pH changes within the capacitor cell are dynamic and cannot be neglected. The onset of these changes begins even at low voltages and from the first cycle. Furthermore, they become especially important for a deep understanding of the maximum voltage abilities of the system. It provides insight into the aging process of electrochemical capacitors for voltage values above the theoretical water decomposition. The strict potential values at which solvent decomposition takes place cannot be calculated and defined beforehand on the basis of the initial electrolyte solution pH measurements. It can only be a primary approximation. The pH in the system is proton, thus, potential-dependent; therefore, the value of this potential is changing depending on the voltage that is maintained by the system. Electrode separation has been shown to have a visible effect on the interpretation of the electrochemical results. Electrolyte decomposition traces can be found even at a voltage lower than the theoretical limit. The GC-MS experiment proved that the products (CO_2_, H_2_O_2_) affect the surface chemistry of the carbon electrodes by inducting the pH changes, which are opposite to those expected from the electrostatic attraction of ions.

The pH at the positive electrode during charging, emerging from the accumulation of OH^–^, initially alkalizes. After the critical potential has exceeded, the OH^–^ ions enter the porosity and the solution in the vicinity of the electrode becomes acidic. These findings were confirmed by the EQCM experiment, where the recorded mass change reflects the adsorption of OH^–^ instead of SO_4_^2–^ ions.

To make aqueous-based capacitors reach high voltage and compete with organic-based capacitors, the consideration of pH maintenance seems to be vital. It is necessary to keep in mind that the trade-off between the energy, resistance, and cycle life is found. The higher the distance between the electrodes, the higher the energy, but also the resistance. As demonstrated, increasing the distance enhances the cycle life. If one wants to obtain a high-power cell, then instead of decreasing the electrode separation and thus resistance, it seems more convenient to use a high conductivity electrolyte. This will allow the same resistance to be maintained. Another issue emerges from system mass, which is highly dependent on the electrolyte amount and its concentration. Certainly, the less electrolyte, the higher gravimetric energy for the system can be obtained. The design of the perfect system should anticipate all of the parameters mentioned.
